# Associations of physical activity with childhood asthma, a population study based on the WHO - health behaviour in school-aged children survey

**DOI:** 10.1186/s40733-018-0042-9

**Published:** 2018-04-30

**Authors:** Lene Lochte, Poul Erik Petersen, Kim G. Nielsen, Anette Andersen, Thomas A. E. Platts-Mills

**Affiliations:** 1 Department of Odontology, University of Copenhagen, 1014 Copenhagen, Denmark; 2 Department of Paediatrics and Adolescent Medicine, Copenhagen University Hospital, Rigshospitalet, 2100 Copenhagen, Denmark; 3 National Institute of Public Health, University of Southern Denmark, 1455 Copenhagen, Denmark; 4 Department of Medicine, Division of Allergy and Clinical Immunology, University of Virginia, Charlottesville, VA 22908 USA

**Keywords:** Ever/or current asthmatic disease, PA, Paediatric, Children, Adolescents, HBSC, School survey

## Abstract

**Background:**

Asthma in paediatric populations is one of the highest public health concerns. In this study of children and adolescents, we hypothesized that low levels of physical activity (PA) would show associations with asthma that vary by asthma outcome. The objective was to assess whether PA was associated with *ever asthma* and/or *current asthma*.

**Methods:**

Analyses were based on 4824 Danish schoolchildren aged 11–15 years old (48.7% boys) participating in the HBSC survey. The study variables were (1) physician-diagnosed asthma (*ever asthma)* and (2) physician-diagnosed asthma plus wheezing and/or physician or hospital consultation for wheezing *(current asthma)*. Associations with PA by gender were analysed with multivariate logistic regression using the “variance covariance (vce) cluster” method.

**Results:**

The prevalence of *ever asthma* was 14.3% (boys) and 12.8% (girls), and that of *current asthma* was 6.8% (boys) and 7.0% (girls). Boys with *current asthma* showed important differences in low and high PA. We found inverse associations between low PA and *ever asthma*, odds ratio [95% confidence interval] male: .55 [.30; .99] and female: .47 [.24; .93], and *current asthma,* male: .27 [.12; .60] (*P linear trend* = .007) and female: .32 [.11; .94].

**Conclusions:**

The lowest activity levels showed significant inverse associations with asthma, regardless of the definition. For boys, the more stringent (*current asthma*) of the two paediatric asthma definitions revealed a significant trend with PA, and the direction of associations shifted to positive as weekly PA increased.

**Electronic supplementary material:**

The online version of this article (10.1186/s40733-018-0042-9) contains supplementary material, which is available to authorized users.

## Background

Asthma in paediatric populations is a serious public health concern worldwide [1]. The associations between asthma and physical activity (PA) may be important for disease prevention and control [2, 3], but reports on the mechanisms behind this relationship are scarce [4]. Asthma is one of the most common lower respiratory diseases in children [5], and its increasing chronic and acute disease burden has caused considerable distress among national health authorities [6]. Similarly, people in most countries perform historically low levels of PA [7], and 80% do not fulfil the current international guidelines for PA [8]. Low PA in children has been proposed as one explanation for the so-called “asthma epidemic” [9].

The severity of paediatric asthma depends on the awareness of the disease condition in the population. The diagnosis of asthma is often made by a physician according to medical history and clinical symptoms [9]. However, the sharp increases in morbidity and mortality have not been explained by alterations in diagnostic practices [10]. Depending on the measurement methods used, asthma severity can influence cardiorespiratory fitness (CRF) [11]. In other paediatric studies, a questionable asthma diagnosis, i.e., with low sensitivity, has not revealed associations between asthma severity and maximal oxygen uptake [12]. Given the worldwide variations in the occurrence of childhood asthma [6], it is often not clear which diagnosis of asthma to apply when analysing population data on children. In observational studies, data are frequently gathered by self-reported methods and rarely include clinical examinations.

Some studies have described associations of asthma in children with low PA [13, 14] or low CRF [15]. PA has been suggested to modify CRF in asthmatic and healthy children [16, 17]; however, asthma in children comprises a variety of symptom phenotypes [18], and effective preventions depend on determining the precise aetiology.

We hypothesized that low levels of PA in 11- to 15-year-olds would show associations with childhood asthma that vary by asthma outcome. Therefore, the objective was to assess whether PA was associated with asthma when defined as *ever asthma* and/or *current asthma* during childhood and adolescence.

## Methods

### Study population and participation rates

We used Danish data that were part of the international World Health Organization (WHO)-coordinated Health Behaviour in School-aged Children (HBSC) survey [19]; the *design* was cross-sectional [20]. The HBSC aimed to increase the understanding of adolescents’ lifestyle and its associations with healthy behaviours [21]. The study used repeated multi-country surveys conducted every four years, primarily in Europe. In Denmark, a survey was conducted six times from 1984 to 2002. The HBSC protocol was standardized (sampling, data collection, compilation) and used self-completed, anonymous HBSC questionnaires administered at schools [22]. PA and asthma were measured for the first time in 1985 and 2002, respectively [21, 23].

The present data were collected from Feb-Apr 2002 and included all students in fifth, seventh, and ninth grade based on a random sample of Danish schools (the mean age in years: 11.6, 13.6, and 15.6, respectively). The 2002 survey comprised 68 schools with 5400 students in 297 classes. Of these, 4981 students (92.2%) were present on the day of data collection, and 4824 students (96.8% of the students present) returned a completed questionnaire.

#### Non-response

There were two sources of non-response: i) schools, classes, and individuals who refused to participate and ii) absence on the data collection day. No follow-up was conducted for absent students. Of the 79 eligible schools, eleven (13.9%) school authorities declined participation. The Danish part of the survey excluded 31 (0.64%) questionnaires due to missing information on gender or inappropriate responses.

### Measurements

Outcome: Based on a validated asthma variable [23], we established two binary asthma definitions: *ever asthma,* defined by a positive response to the question “Has the doctor ever told you that you have asthma?”, and *current asthma,* defined by a positive response to the question “Has the doctor ever told you that you have asthma?” plus a positive response to either “In the last 12 months, have you had episodes of wheezing?” or “In the last 12 months, have you had a consultation for wheezing by a doctor or in a hospital?”

The exposure was PA, which was recorded as responses to the following: “Outside of school hours, how many hours a week do you participate in sports or exercise that make you out of breath or sweaty?” There were six response options reported in hours (h) per week (wk) as follows: “0”, “.5”, “1”, “2–3”, “4–6”, “> = 7”. The PA question on the HBSC survey [21] adhered to a validated standard based on Australian schoolchildren [24]. The following covariates were assessed: grade (fifth, seventh, ninth), self-rated health (poor, good, excellent), body mass index (BMI) (13–15, 16–18, 19–21, > 21, defined as reported body weight in kilograms (kg) divided by reported height in metres (m) squared (kg•m^− 2^)), and menarche (yes/no).

### Statistical methods

Frequencies (%) and means (±2SD) were calculated. For the statistical evaluation, we applied the Chi-square test of homogeneity (Pearson’s χ^2^). Spearman’s correlation coefficient (rho) was assessed to express the correlations between physician-diagnosed asthma and the asthma symptom variables used to define the asthma outcomes in the children investigated.

Univariate analyses produced crude odds ratios (ORs) and 95% confidence intervals (CIs) and assisted the identification of potential confounding variables (Additional file 1). A priori, we selected possible confounding variables using known influential variables and evidence from our univariate analyses. We applied the following criterion for a variable to possibly confound a given association: The variable (or groups of variables) demonstrated associations with the exposure, the outcome or both [25]. Additionally, Mantel-Haenszel stratification and likelihood ratio tests confirmed that BMI substantially influenced the associations under study. Formal tests of effect modification by self-rated health did not provide any response in these data (*P* for interaction, male = 0.2004, female = 0.3912). Hence, the covariates adjusted for were as follows: grade and self-rated health (both genders), BMI (boys) (ever asthma only) and menarche (girls). We considered our analytical models along these lines and built them accordingly.

All analyses were conducted separately for males and females. For the outcome, we used two different definitions of childhood asthma. The results for *ever asthma* and *current asthma* are presented in this paper.

In the multivariate logistic regression models, we used the “variance covariance (vce) cluster” method, which calculated robust standard errors using schools as clusters. This approach allowed for correlated observations, e.g., potential effects of clusters induced by the HBSC sampling method. Hence, we estimated the associations, i.e., ORs (95% CIs), for childhood asthma and PA exposure. High PA (7^+^ hours per week) served as the reference, and we estimated the probability for linear trend. The selection of the reference group was based on tertiles of our exposure variable, and we adopted the criterion that more than 50% of the exposed should be in this group [26]. We used STATA™ (v.12.1) (Stata Corp, College Station, TX, USA). All tests of significance were two-tailed, and *P* <  0.05 was considered statistically significant, though strength against the null hypothesis was regarded as superior to the critical value set [27].

## Results

Characteristics of the study participants are depicted in Table 1. A total of 4824 schoolchildren (48.7% boys) participated. Table 1 shows the reported asthma symptoms (wheezing and doctor/hospital consultation for wheezing) for males and females. The prevalence of *ever asthma* was 14.3% for boys and 12.8% for girls; the *current asthma* prevalence was 6.8% in boys and 7.0% in girls. Table 2 shows the reported PA categories (low to high) distributed by male and female gender and according to *ever asthma* (Table 2) and *current asthma* (Table 3). Boys with *current asthma* exhibited important differences in low and high PA. Details of the univariate associations for *ever asthma* and *current asthma* are presented in Additional file 1. Additional file 2 presents the correlations (Spearman’s rho, r) for physician-diagnosed asthma and the asthma symptom variables used to define the respective outcomes, i.e., *ever asthma* and *current asthma*. Table 4 and Table 5 present the crude and adjusted associations, OR and 95% CI, for PA (0–7^+^ hours per week) and *ever asthma* and *current asthma*, respectively. After adjustments, we found inverse associations (i.e., possible protective associations) between PA and asthma using both definitions. With *ever asthma*, inverse associations were present in boys reporting zero hours of PA per week and for girls reporting 0–1 h of PA per week, with risk reductions of 45% and 53% in males and females, respectively (Table 4). For *current asthma,* inverse associations were present for boys reporting zero hours of PA per week and for girls reporting .5 h of PA per week, with risk reductions of 73% and 68% in males and females, respectively (Table 5). For *current asthma*, there was a significant trend (*P* = .007) in boys (Table 5), and the associations shifted in a positive direction as weekly PA increased. There was evidence of an attenuation of effects for *ever* (Table 4) and *current asthma* (Table 5) in both males and females after adjusting for possible confounders.

**Table 1:** Characteristics of all study participants (%) by gender

	Boys (n)	Girls (n)	*P*
	48.7 (2348)	51.3 (2476)	
Participants by grade			= 0.462
5	35.6 (836)	37.0 (916)	
7	34.4 (807)	32.8 (812)	
9	30.0 (705)	30.2 (748)	
Wheeze^AS^	14.2 (333)	17.2 (426)	= 0.003
Mis	1.3 (31)	1.6 (40)	
Doctor /or hospital consultation for wheeze^AS^	6.4 (151)	7.0 (172)	= 0.481
Mis	1.0 (23)	0.9 (21)	
Ever asthma	14.3 (336)	12.8 (316)	= 0.120
Mis	0.8 (18)	0.9 (22)	
Current asthma	6.8 (160)	7.0 (173)	= 0.806
Mis	2.3 (53)	2.3 (58)	
Low PA (0-1 h/week)	24.5 (568)	33.1 (810)	< 0.001
Mis	1.1 (25)	1.1 (28)	

*AS *Asthma symptom past 12 months, *H* Hour, *Mis* Missing, *N* Number, *Ns* Non-significant, *P* Probability of difference between genders (Pearson chi^2^), *PA* Physical activity

**Table 2:** Distribution (%) of weekly PA according to *ever* *asthma* for participating boys and girls

PA (h per wk.)	Boys (n)	Girls (n)	*P* ^*a*^
Low (0–1)	22.2 (74)	29.5 (93)	= 0.408
Mod (2–3)	28.7 (96)	34.3 (108)	= 0.490
High (4–7+)	49.1 (164)	36.2 (114)	= 0.498
*P* ^*b*^	= 0.524	= 0.323	

*H *Hour/s, *Mod* Moderate, *N* Number, *P* Probability of difference (Pearson chi^2^) between (a) genders, (b) PA categories, *PA* Physical activity, *Wk* Week

Overall Pearson chi^2^: *P* = 0.003

**Table 3:** Distribution (%) of weekly PA according to *current asthma* for participating boys and girls

PA (h per wk.)	Boys (n)	Girls (n)	*P* ^*a*^
Low (0–1)	16.4 (26)	27.2 (47)	= 0.296
Mod (2–3)	28.3 (45)	36.4 (63)	= 0.509
High (4–7+)	55.4 (88)	36.4 (63)	= 0.749
*P* ^b^	= 0.025	= 0.229	

*H* Hour/s, *Mod* Moderate, *N* Number, *P* Probability of difference (Pearson chi^2^) between (a) genders, (b) PA categories, *PA* Physical activity, *Wk* Week

Overall Pearson chi^2^: *P* = 0.002

**Table 4:** Crude and adjusted associations for *ever asthma* i.e. Asthma Definition [1] by physical activity categorised (0-7^+^ hours per week), odds ratio and 95% confidence interval

	Boys	Girls
“Ever Asthma” (%, n, total)	(14.4, 336, 2330)	(12.9, 316, 2454)
PA (all categories) (n)	2323	2448
0 h/wk. (%, n)	9.3, 216	11.2, 275
Crude OR (95% CI)^a^	0.70 (0.40; 1.22)^*P* = 0.207^	0.79 (0.52; 1.19)^*P* = 0.255^
Adjusted OR (95% CI)^b^	0.55 (0.30; 0.99)^*P* = 0.046^	0.62 (0.40; 0.97)^*P* = 0.034^
0.5 h/wk. (%, n)	5.6, 129	6.3, 153
Crude OR (95% CI)^a^	0.94 (0.50; 1.77)^*P* = 0.840^	0.60 (0.32; 1.12)^*P* = 0.109^
Adjusted OR (95% CI)^b^	0.73 (0.35; 1.53)^*P* = 0.409^	0.47 (0.24; 0.93)^*P* = 0.030^
1 h/wk. (%, n)	9.6, 223	15.6, 382
Crude OR (95% CI)^a^	1.16 (0.70; 1.91)^*P* = 0.576^	0.69 (0.43; 1.12)^*P* = 0.136^
Adjusted OR (95% CI)^b^	0.96 (0.53; 1.74)^*P* = 0.897^	0.60 (0.37; 0.98)^*P* = 0.039^
2–3 h/wk. (%, n)	28.6, 664	33.5, 819
Crude OR (95% CI)^a^	1.05 (0.77; 1.43)^*P* = 0.746^	0.83 (0.57; 1.21)^*P* = 0.332^
Adjusted OR (95% CI)^b^	1.00 (0.70; 1.42)^*P* = 0.982^	0.73 (0.50; 1.07)^*P* = 0.104^
4–6 h/wk. (%, n)	27.2, 631	22.4, 549
Crude OR (95% CI)^a^	1.17 (0.88; 1.56)^*P* = 0.270^	0.82 (0.53; 1.28)^*P* = 0.383^
Adjusted OR (95% CI)^b^	1.17 (0.84; 1.62)^*P* = 0.353^	0.72 (0.45; 1.15)^*P* = 0.171^
7+ h/wk. (%, n)	19.8, 460	11.0, 270
Ref	1.00	1.00
*P* linear trend	= 0.235	= 0.189

*HBSC* Health Behaviour in School-aged Children, *H* Hour/s, *Mod* Moderate, *Ref* Reference category, *WHO* World Health Organization, *Wk* Week

^a^Univariate analysis i.e. crude effect of PA (n boys: 2311; n girls: 2429)

^b^Multivariate analysis i.e. “full confounder adjustment model” (n boys: 2058; n girls: 2347)

Adjustments (male: Grade, self-rated health, BMI; female: Grade, self-rated health, menarche)

*P*: Chi^2^

**Table 5:** Crude and adjusted associations for *current asthma* i.e. Asthma Definition [2] by physical activity categorised (0-7^+^ hours per week), odds ratio and 95% confidence interval

	Boys	Girls
“Current Asthma” (%, n, total)	(7.0, 160, 2295)	(7.2, 173, 2418)
PA (all groups) (n)	2323	2448
0 h/wk. (%, n)	9.3, 216	11.2, 275
Crude OR (95% CI)^a^	0.45 (0.20; 1.03)^*P* = 0.060^	0.81 (0.41; 1.57)^*P* = 0.528^
Adjusted OR (95% CI)^b^	0.27 (0.12; 0.60)^*P* = 0.002^	0.56 (0.28; 1.14)^*P* = 0.108^
0.5 h/wk. (%, n)	5.6, 129	6.3, 153
Crude OR (95% CI)^a^	0.76 (0.33; 1.78)^*P* = 0.529^	0.52 (0.21; 1.32)^*P* = 0.171^
Adjusted OR (95% CI)^b^	0.48 (0.20; 1.16)^*P* = 0.102^	0.32 (0.11; 0.94)^*P* = 0.039^
1 h/wk. (%, n)	9.6, 223	15.6, 382
Crude OR (95% CI)^a^	0.77 (0.40; 1.46)^*P* = 0.417^	0.63 (0.31; 1.27)^*P* = 0.198^
Adjusted OR (95% CI)^b^	0.55 (0.28; 1.06)^*P* = 0.073^	0.52 (0.26; 1.01)^*P* = 0.055^
2–3 h/wk. (%, n)	28.6, 664	33.5, 819
Crude OR (95% CI)^a^	0.99 (0.62; 1.58)^*P* = 0.981^	0.91 (0.51; 1.63)^*P* = 0.747^
Adjusted OR (95% CI)^b^	0.76 (0.46; 1.27)^*P* = 0.299^	0.82 (0.46; 1.46)^*P* = 0.496^
4–6 h/wk. (%, n)	27.2, 631	22.4, 549
Crude OR (95% CI)^a^	1.34 (0.83; 2.15)^*P* = 0.231^	0.85 (0.44; 1.64)^*P* = 0.631^
Adjusted OR (95% CI)^b^	1.21 (0.72; 2.03)^*P* = 0.479^	0.77 (0.39; 1.51)^*P* = 0.444^
7+ h/wk. (%, n)	19.8, 460	11.0, 270
Ref	1.00	1.00
*P* linear trend	= 0.007	= 0.229

*HBSC* Health Behaviour in School-aged Children, *H* Hour/s, *Mod* Moderate, *Ref* Reference category, *WHO* World Health Organization, *Wk* Week

^a^Univariate analysis i.e. crude effect of PA (n boys: 2277; n girls: 2397)

^b^Multivariate analysis i.e. “full confounder adjustment model” (n boys: 2265; n girls: 2319)

Adjustments (male: Grade, self-rated health; female: Grade, self-rated health, menarche)

*P*: Chi^2^

## Discussion

In this population of schoolchildren, our key finding from the cross-sectional data was that low PA showed significant inverse associations, regardless of asthma diagnosis. In addition, high PA among boys demonstrated positive effects, suggesting elevated asthma risks. We confirmed this finding by identifying a positive dose-response for boys with *current asthma*.

Our study sample was nationally representative, but our results can be compared with those of international HBSC collaborators [23] as well. Our results are consistent with those of other authors in addition to the HBSC. For example, although the findings were not reported separately for boys and girls, current asthma reports show null effects for lower activity levels and increased risk with PA exceeding 7 h per week [28]. Although asthmatic children and adolescents can be as physically active as healthy children [28, 29], they may still demonstrate lower CRF [30]. The current study determined that PA is a behavioural factor that varies by context and time, whereas CRF is genetically determined and therefore tends to remain stable over time [31]. Additionally, our results seem to support a previous study investigating sports frequency and incident wheezing in paediatric populations [14]. That study found inverse associations of higher activity in boys and girls [14], and based on the validated PA measure (“active”: one hour or more per week) [24] used in the HBSC survey, it is plausible that the activity frequency reported by our colleagues [14] is comparable to our results for 1–3 h of PA per week. In addition, the “leisure-type PA outside school hours” assessed in our HBSC data may be similar to the swimming and running that our colleagues [14] studied. The current study did not measure CRF, and thus we cannot rule out that certain weekly running programmes may have decreased exercise-induced bronchoconstriction [32] and subsequently protected against wheezing.

The selection of participating schoolchildren by “cluster sampling” may introduce selection bias. To account for this design effect, we adjusted our final analyses to account for non-independent variables. A computer–assisted random selection of the eligible schools was performed, and we reviewed the initial non-responses in eleven schools. According to the study protocol, the reasons for refusing participation were within the frequently reported categories, for example, administrative overload or recent participation in another survey. The participating schools were both private and public, and social class did not influence our findings; in contrast to the International Study of Asthma and Allergies in Childhood (ISAAC)‘s urban records [33], our HBSC data in mixed rural and urban contexts likely exhibited substantial variability, which improved the accuracy of the measurements. Thus, the factors discussed seem to support a sound representativeness of our data.

The attenuation of effects that we observed from the crude to the adjusted results is consistent with others’ observations [34]. Some previous studies also found positive associations between low PA and childhood asthma but with certain restrictions. For example, the results of a study with New Zealand children, who are sometimes considered comparable to Scandinavian populations, indicated inverse effects in children but not in adolescents [13]. Although our two studies both investigated vigorous activities, the asthma diagnoses varied. In contrast to the HBSC, ISAAC [13] included parental assistance for the youngest children. We adjusted for grade to represent the age of the students, as this factor confounded the associations. However, other colleagues noted that, compared to the children, the parents of asthmatic children reported more PA [35]. Moreover, the children we studied could have been providing information about, e.g., a viral infection [12, 33]; based on the questions about asthma that involved contact with physicians or the hospital, the risk of non-random misclassification seems low, but this possibility could have been assessed quantitatively using sensitivity analyses [36]. Additionally, our results for the male participants seem to agree with recent findings that demonstrate positive associations between high PA and asthma [37]. These authors [37] studied club sports, and it is likely that our assessment of after-school PA represent organized activities as well; therefore, our two studies may already be comparable with respect to basic data.

An advantage of the current study is that the PA variable had been validated previously demonstrating acceptable validity and reliability and that a relevant reference was used, as the Australian population is often comparable to European populations [24]. Our findings for PA may align with previous results, which suggest that season of the year and low PA are associated [38]. Our data were collected in the spring; in Denmark, this time of the year typically reflects an increase in children’s participation in outdoor PA. The partially comparable pattern of PA for boys and girls in the reported after-school activities seem consistent with findings measured using accelerometry [39]; it is clear that the overall variances in these two studies pertained to the high-intensity leisure-time PA performed by the participating boys. Overall, the two asthma outcomes elucidated how asthma in young populations can be expressed using epidemiological data. One advantage of the current findings is the fairly consistent results of the broader and the more restrictive outcome. While the definition of *ever asthma* is well-established, *current asthma* has been studied using varying definitions [40, 41]. Earlier comparisons of ever asthma and wheezing show higher prevalence of wheezing, although wheezing still showed reasonable sensitivity and specificity [33]. *Current asthma* is likely to represent a more severe variation of asthma symptoms, as the prevalence using this definition was nearly half that of *ever asthma*.

The self-reported nature of our data may be a limitation. The type of report used is highly important to asthma outcomes, which are based on children’s ability to recall that a physician told them that they have asthma. Although we identified a relatively high prevalence of *ever asthma*, this rate was consistent with the global [6] and HBSC prevalence [42]. In fact, the study that validated the HBSC asthma questions [23] found that significantly more parents than children reported “diagnosed as having asthma”. In our study, we cannot exclude that some slight overreporting of both *ever* and *current asthma* may have overestimated the prevalence obtained. Additionally, in our analyses, we did not distinguish between younger and older children, but the youngest children in our sample were 11 years of age; hence, as generally seen in the HBSC surveys, there could be less accurate reporting of factors 12 months prior to the survey, particularly by the youngest children. The ISAAC contributed in part to the HBSC asthma questions, but we propose that any discrepancy in reporting by age groups of those 11 (HBSC) and 13 (ISAAC) years would have limited impact on our results. The HBSC asthma questions are based on the Brief Pediatric Asthma Screening (BPAS) questionnaire, which has reasonable sensitivity and only minor misclassification [23]. These findings support our argument that the present HBSC data are valid.

The severity of paediatric asthma studied was indirectly included in the asthma symptom questions. In addition to wheezing, we used a second symptom, “doctor or hospital consultation for wheezing”. This approach is standard in epidemiological studies of asthma in children [41]. In the original scale validation of the HBSC asthma questions, these two symptoms both obtained high scores [23]. While type of PA was not specified in the HBSC questionnaire, the PA question asked about activities that led to “sweating” or “getting out of breath”. In line with earlier findings [43], it is possible that some of the children in our study had mild or moderate asthma, as high PA was well represented, particularly by the boys. We cannot directly distinguish the asthmogeneity of the activities reported by the children, but the HBSC protocol states that PA should be of the “moderate-to-vigorous” intensity. The HBSC asthma questions do not ask about participants’ medical treatment for asthma, and thus the proxy we used was “consultation for wheezing by a doctor or in a hospital” to represent any exacerbation of respiratory symptoms that required medical attention. As this question asked about a hospital episode, the risk of recall bias was considered low. Asthmatic children may need medical treatment to participate in the high-intensity sports reported. As we used child-reported data as opposed to parental reports, the risk of systematic overreporting was not apparent. The lack of a reference time [24], which is equally important to self-reported asthma and the PA of the children surveyed in HBSC, may be a limitation. Thus, memory cues for PA, for example, “within a fortnight” or “on weekdays”, likely could have improved the accuracy of the self-reports provided by the children. The limitations associated with children’s self-reported PA are widely recognised [44]; although our PA data do not provide information on all PA dimensions, the pattern of our results for PA and asthma from the current Danish HBSC study aligns with that of other European countries [7]. We observed some indications of boys with asthma being more physically active on a weekly basis than girls with asthma. This finding further suggests that boys and girls with asthmatic diseases and those in the background population follow similar patterns with regard to their PA. Meanwhile, our data for the asthmatic boys and girls clearly showed that two-thirds reported activity levels above the recommended European standard of 60 min per day.

Finally, the questionnaire data revealed no obvious gender differences for any of the asthma types. This finding is in line with earlier findings—at least for low PA levels [45]; however, supplementary activity monitoring at the leisure settings may have improved our design and the ability of the methods to detect any gender discrepancy.

The current data were cross-sectional, and caution is thus warranted in interpreting our findings as cause-and-effect. We did not ascertain the directions of the associations, and our results could be subject to reverse causation. However, in other cross-sectional designs, our colleagues [40, 46] analysed the opposite order of events, i.e., childhood asthma was defined as the exposure. One of these studies [40] demonstrated an association between severe asthma in children and low PA. This association may support our findings for some participants. The other study [46] demonstrated that children with asthma had a good chance of performing moderate activity, which is consistent with our observations; nonetheless, the temporal sequence of our results cannot be accounted for by the present point observations. Furthermore, the HBSC design ensured the total anonymity of participants, which precluded us from tracking our participants over time. While we were unable to detect the future direction of the associations, overall, cross-sectional studies of asthma in children use heterogeneous methodologies [47], and thus future comparisons highly depend on the application of a systematic approach.

## Conclusions

The lowest PA levels showed significant inverse associations with asthma, regardless of the definition. For boys, the more stringent (*current asthma*) of the two paediatric asthma definitions revealed a significant trend with PA, and the associations shifted in a positive direction as weekly PA increased.

## Additional files

Additional file 1: Univariate analyses. Presents univariate analyses for PA, covariates, Ever Asthma (Tables 15, 16); PA, covariates, Current Asthma (Tables 15^1^, 16^1^); covariates, PA (Table 17). (PDF 133 kb)

Additional file 2: Correlation matrix. Presents correlations for asthma variables. (DOCX 23 kb)

## Abbreviations

BMI: Body mass index
BPAS: Brief Pediatric Asthma Screening
CRF: Cardiorespiratory fitness
HBSC: Health Behaviour in School-aged Children
ISAAC: International Study of Asthma and Allergies in Childhood
PA: Physical activity
WHO: World Health Organization
