# Supplementary material for: Associations of physical activity with childhood asthma, a population study based on the WHO - health behaviour in school-aged children survey
Source: Asthma Res Pract. 2018 Apr 30;4:6. doi: 10.1186/s40733-018-0042-9 (PMC5925826; doi:10.1186/s40733-018-0042-9)
Supplement: Supplementary file 1 — Univariate analyses. Presents univariate analyses for PA, covariates, Ever Asthma (Tables 15, 16); PA, covariates, Current Asthma (Tables 151, 161); covariates, PA (Table 17). (PDF 133 kb) [file 40733_2018_42_MOESM1_ESM.pdf]

Additional file 1

Univariate analyses (performed prior to multivariate logistic regression vce analyses)

Analysis of outcome Asthma Definition [1]: ***Ever Asthma*** i.e. "yes" to question "has a doctor ever told you that you have asthma?"

Exposure PA versus outcome Ever Asthma

Table 15: Unadjusted odds ratio (OR) with 95% confidence interval (CI) for ever asthma by physical activity (PA) (hrs/wk) in boys and girls.

	Boys			Girls		
Covariate¶	OR (95% CI)	*P*	N	OR (95% CI)	*P*	N
PA	1.06 (0.97; 1.14)	=0.1859	2311	1.06 (0.98; 1.15)	=0.1677	2429

Hrs: Hours; Ns: Non-significant; Wk: Week; P: Chi^2 ¶ Covariate PA analysed as continuous

Covariates versus outcome Ever Asthma

Table 16: Unadjusted odds ratio (OR) with 95% confidence interval (CI) for ever asthma by individual covariates in boys and girls.

	Boys			Girls		
Covariate¶	OR (95% CI)	*P*	N	OR (95% CI)	*P*	N
Age	1.03 (0.96; 1.11)	=0.4271	2280	1.07 (1.00; 1.15)	=0.0522	2416
Grade	1.04 (0.90; 1.19	=0.6372	2330	1.14 (0.99; 1.32)	=0.0738	2454
Month of birth	0.99 (0.96; 1.03)	=0.6148	2309	0.99 (0.96; 1.03)	=0.5699	2443
Siblings	0.85 (0.63; 1.13)	=0.2637	2330	0.94 (0.70; 1.27)	=0.6908	2454
Smoking student	0.98 (0.84; 1.15)	=0.8350	2292	0.94 (0.81; 1.09)	=0.4103	2429
Smoking maternal	0.87 (0.78; 0.97)	=0.0140	2230	0.89 (0.79; 1.01)	=0.0626	2384
Height	1.00 (0.99; 1.01)	=0.3933	2159	1.00 (0.99; 1.01)	=0.9363	2327
Weight per 10kg change	1.05 (0.96; 1.14)	=0.2711	2157	0.99 (0.89; 1.11)	=0.8731	2257
BMI	1.21 (1.07; 1.38)	=0.0024	2078	1.00 (0.88; 1.14)	=0.9593	2206
Social class	0.99 (0.92; 1.07)	=0.7973	2300	1.05 (0.97; 1.14)	=0.2346	2433
Self-rated health	1.85 (1.57; 2.19)	<0.001	2315	1.68 (1.41; 1.99)	<0.001	2441
Menarche				1.43 (1.11; 1.85)	=0.0057	2383

Ns: Non-significant; P: Chi^2 ¶ All covariates analysed as continuous

Analysis of outcome Asthma Definition [2]: ***Current Asthma*** i.e. “Ever asthma yes” + “yes” to either of two current asthma symptoms i.e. “In the last 12 months, have you had episodes of wheeze?” /or “In the last 12 months, have you had a consultation for wheeze by a doctor or in an emergency room?”

Exposure PA versus outcome Current Asthma

Table 15[1]: Unadjusted odds ratio (OR) with 95% confidence interval (CI) for current asthma by physical activity (PA) (hrs/wk) in boys and girls.

	Boys			Girls		
Covariate¶	OR (95% CI)	*P*	N	OR (95% CI)	*P*	N
PA	1.16 (1.03; 1.30)	=0.0117	2277	1.08 (0.96; 1.20)	=0.1933	2397

Hrs: Hours; Ns: Non-significant; Wk: Week; P: Chi^2 ¶ Covariate PA analysed as continuous

Covariates versus outcome Current Asthma

Table 16[1]: Unadjusted odds ratio (OR) with 95% confidence interval (CI) for current asthma by individual covariates in boys and girls.

	Boys			Girls		
Covariate¶	OR (95% CI)	*P*	N	OR (95% CI)	*P*	N
Age	1.03 (0.94; 1.14)	=0.4966	2246	1.13 (1.03; 1.25)	=0.0088	2381
Grade	1.05 (0.86; 1.28)	=0.6328	2295	1.27 (1.05; 1.54)	=0.0131	2418
Month of birth	1.00 (0.95; 1.05)	=0.9060	2274	1.01 (0.97; 1.06)	=0.6377	2407
Siblings	0.73 (0.50; 1.07)	=0.1153	2295	1.18 (0.78; 1.79)	=0.4167	2418
Smoking student	0.98 (0.79; 1.22)	=0.8782	2260	0.86 (0.72; 1.02)	=0.1026	2397
Smoking maternal	1.03 (0.88; 1.20)	=0.7125	2198	0.83 (0.71; 0.97)	=0.0185	2352
Height	1.00 (0.98; 1.01)	=0.4579	2130	1.01 (0.99; 1.03)	=0.3230	2297
Weight per 10kg change	1.00 (0.89; 1.13)	=0.9976	2129	1.03 (0.89; 1.19)	=0.6962	2230
BMI	1.23 (1.03; 1.46)	=0.0198	2053	1.02 (0.87; 1.20)	=0.8223	2180
Social class	0.93 (0.84; 1.04)	=0.2177	2267	1.06 (0.95; 1.17)	=0.3029	2397
Self-rated health	2.57 (2.04; 3.22)	<0.001	2282	2.36 (1.88; 2.95)	<0.001	2407
Menarche				1.81 (1.27; 2.58)	=0.0007	2350

Ns: Non-significant; P: Chi^2 ¶ All covariates analysed as continuous except menarche (results are from categorized variable)

Covariates versus physical activity (PA) (binary)

Table 17: Unadjusted odds ratio (OR) with 95% confidence interval (CI) for PA (4-7$^+$ hrs/wk) by individual covariates in boys and girls.

	Boys			Girls		
Covariate¶	OR (95% CI)	*P*	N	OR (95% CI)	*P*	N
Age	1.01 (0.96; 1.06)	=0.7947	2273	1.05 (1.00; 1.10)	=0.0769	2410
Grade	1.03 (0.94; 1.14)	=0.5091	2323	1.13 (1.02; 1.26)	=0.0169	2448
Month of birth	0.99 (0.96; 1.01)	=0.2662	2302	1.00 (0.97; 1.02)	=0.8885	2437
Siblings	1.14 (0.93; 1.41)	=0.2150	2323	1.30 (1.04; 1.62)	=0.0194	2448
Smoking student	1.18 (1.06; 1.33)	=0.0034	2287	1.04 (0.93; 1.17)	=0.4534	2424
Smoking maternal	1.00 (0.92; 1.08)	=0.9561	2227	1.10 (1.01; 1.20)	=0.0285	2377
Height	1.00 (1.00; 1.01)	=0.5934	2159	1.01 (1.00; 1.02)	=0.0024	2331
Weight per 10 kg change	1.00 (0.94; 1.06)	=0.9885	2156	1.09 (1.01; 1.18)	=0.0288	2265
BMI	0.95 (0.87; 1.04)	=0.2943	2078	1.02 (0.94; 1.12)	=0.6068	2215
Social class	0.93 (0.88; 0.98)	=0.0065	2294	0.84 (0.79; 0.89)	<0.001	2427
Self-rated health	0.56 (0.49; 0.63)	<0.001	2310	0.80 (0.71; 0.91	=0.0005	2430
Menarche				1.25 (1.05; 1.50)	=0.0117	2376

Hrs: Hours; Ns: Non-significant; Wk: Week; P: Chi^2 ¶ All covariates analysed as continuous
