# Supplementary material for: Associations of physical activity with childhood asthma, a population study based on the WHO - health behaviour in school-aged children survey
Source: Asthma Res Pract. 2018 Apr 30;4:6. doi: 10.1186/s40733-018-0042-9 (PMC5925826; doi:10.1186/s40733-018-0042-9)
Supplement: Supplementary file 2 — Correlation matrix. Presents correlations for asthma variables. (DOCX 23 kb) [file 40733_2018_42_MOESM2_ESM.docx]

**Additional file 2**

Correlation matrice (Spearman’s *r^P^*, n) for physician diagnosed asthma and the asthma symptom variables used to define the respective outcomes *ever asthma* (asthma definition [1]) and *current asthma* (asthma definition [2]).

|  | **Physician diagnosed asthma**  **A.** | **Asthma symptom**  **wz^#^**  **B.** | **Asthma symptom**  **physician or emergency room consultation for wz^#^**  **C.** |
| --- | --- | --- | --- |
| **A.** | 1.0 |  |  |
| **B.** | 0.33^***^, 4732 | 1.0 |  |
| **C.** | 0.24^***^, 4758 | 0.29^***^, 4732 | 1.0 |

N: Number; P: Probability for Spearman’s correlation coefficient rho; r: rho; wz: Wheeze

^#^: Reported for last 12 months; ^***^: *P*<0.001
